# 

**Published:** 1888-06

**Authors:** 


NOTICE.

*All articles for publication, books for review, business communications, etc., must be sent to Editors, 1123 Spruce Street, Philadelphia.*

*Subscribers will please notice that this Journal will be sent until arrears are paid and it is ordered discontinued.*

PHILADELPHIA:

